# Supplementary material for: Pediatric Health Access and Private Medical Insurance: Based on the Ecology of Medical Care in Korea
Source: Children (Basel). 2022 Jul 22;9(8):1101. doi: 10.3390/children9081101 (PMC9330897; doi:10.3390/children9081101)
Supplement: Supplementary file 1 [file children-09-01101-s001.zip › children-1812334-supplementary.pdf]

Supplementary materials

Table S1. Types of doctor-diagnosed diseases reported from 677 individuals

Types of disease	Number (%)	Types of disease	Number (%)
Herpes zoster	2 (0.30)	Chronic sinusitis	18 (2.66)
Chronic viral hepatitis	2 (0.30)	Unspecified chronic bronchitis	2 (0.30)
Dermatophytosis	3 (0.44)	Asthma	12 (1.77)
Other malignant neoplasms of skin	1 (0.15)	Abscess of lung and mediastinum	1 (0.15)
Other benign neoplasms of connective and other soft tissue	2 (0.30)	Dental caries	13 (1.92)
Benign neoplasm of ovary	1 (0.15)	Dentofacial anomalies	15 (2.22)
Benign neoplasm of other and unspecified sites	1 (0.15)	Gastritis and duodenitis	1 (0.15)
Other anaemias	3 (0.44)	Other functional intestinal disorders	1 (0.15)
Other coagulation defects	1 (0.15)	Fissure and fistula of anal and rectal regions	1 (0.15)
Purpura and other haemorrhagic conditions	1 (0.15)	Atopic dermatitis	75 (11.08)
Other hypothyroidism	1 (0.15)	Allergic contact dermatitis	10 (1.48)
Thyrotoxicosis	1 (0.15)	Unspecified contact dermatitis	4 (0.59)
Disorders of puberty, NEC	8 (1.18)	Dermatitis due to substances taken internally	2 (0.30)
Depressive episode	3 (0.44)	Urticaria	1 (0.15)
Other anxiety disorders	1 (0.15)	Acne	7 (1.03)
Specific developmental disorders of scholastic skills	2 (0.30)	Vitiligo	5 (0.74)
Pervasive developmental disorders	4 (0.59)	Polyarteritis nodosa and related conditions	1 (0.15)
Hyperkinetic disorders	11 (1.62)	Scoliosis	9 (1.33)
Tic disorders	2 (0.30)	Dorsalgia	1 (0.15)
Dystonia	1 (0.15)	Other bursopathies	1 (0.15)
Epilepsy	5 (0.74)	Unspecified nephritic syndrome	1 (0.15)
Other disorders of brain	1 (0.15)	Obstructive and reflux uropathy	2 (0.30)
Other disorders of eyelid	1 (0.15)	Other disorders of kidney and ureter, NEC	1 (0.15)
Disorders of lacrimal system	1 (0.15)	Benign mammary dysplasia	1 (0.15)
Keratitis	1 (0.15)	Noninflammatory disorders of ovary, fallopian tube and broad ligament	1 (0.15)
Other strabismus	13 (1.92)	Microcephaly	2 (0.30)
Disorders of refraction and accommodation	15 (2.22)	Congenital hydrocephalus	1 (0.15)
Visual disturbances	4 (0.59)	Spina bifida	1 (0.15)
Other disorders of eye and adnexa	1 (0.15)	Congenital malformations of cardiac chambers and connections	1 (0.15)
Suppurative and unspecified otitis media	14 (2.07)	Congenital malformation of cardiac septa	4 (0.59)
Conductive and sensorineural hearing loss	1 (0.15)	Other congenital malformations of heart	2 (0.30)
Other hearing loss	1 (0.15)	Congenital malformations of great veins	1 (0.15)
Nonrheumatic mitral valve disorders	1 (0.15)	Other congenital malformations of limbs	1 (0.15)
Other conduction disorders	1 (0.15)	Other congenital malformations of skin	1 (0.15)
Paroxysmal tachycardia	1 (0.15)	Down's syndrome	1 (0.15)
Complications and ill-defined descriptions of heart disease	2 (0.30)	Turner's syndrome	1 (0.15)
Other cerebrovascular disease	1 (0.15)	Pain associated with micturition	1 (0.15)
Diseases of capillaries	1 (0.15)	Polyuria	1 (0.15)
Acute tonsilitis	1 (0.15)	Dizziness and giddiness	1 (0.15)
Vasomotor and allergic rhinitis	330 (48 .74)	Convulsions, NEC	1 (0.15)
Chronic rhinitis, nasopharyngitis and pharyngitis	23 (3.40)	Lack of expected normal physiological development	1 (0.15)
